# Evaluation of recombinant porin (rOmp2a) protein as a potential antigen candidate for serodiagnosis of Human Brucellosis

**DOI:** 10.1186/s12879-017-2588-1

**Published:** 2017-07-11

**Authors:** Prachi Pathak, Ashu Kumar, Duraipandian Thavaselvam

**Affiliations:** Defence Research and Development Establishment, Jhansi Road, Gwalior, 474002 India

**Keywords:** Omp2a, Porin, iELISA

## Abstract

**Background:**

Brucellosis is an important zoonotic disease caused by different *Brucella* species and human brucellosis is commonly prevalent in different states of India. Among various *Brucella* species, *B. melitensis* is most pathogenic to human and included as category B biothreat which can cause infection through aerosol, cut, wounds in skin and contact with infected animals. The diagnosis of human brucellosis is very important for proper treatment and management of disease as there is no vaccine available for human use. The present study was designed to clone, express and purify immunodominant recombinant omp2a (rOmp2a) porin protein of *B. melitensis* and to evaluate this new antigen candidate for specific serodiagnosis of human brucellosis by highly sensitive iELISA (indirect enzyme linked immunosorbent assay).

**Method:**

*Omp2a* gene of *B. melitensis* 16 M strain was cloned and expressed in pET-SUMO expression system. The recombinant protein was purified under denaturing conditions using 8 M urea. The purified recombinant protein was confirmed by western blotting by reacting with anti-HIS antibody. The sero-reactivity of the recombinant protein was also checked by reacting with antisera of experimentally infected mice with *B. melitensis* 16 M at different time points. Serodiagnostic potential of recombinant porin antigen was tested against 185 clinical serum samples collected from regions endemic to brucellosis in southern part of India by iELISA. The samples were grouped into five groups. Group 1 contained cultured confirmed positive serum samples of brucellosis (*n* = 15), group 2 contained sera samples from positive cases of brucellosis previously tested by conventional methods of RBPT (*n* = 28) and STAT (*n* = 26), group 3 contained sera samples negative by RBPT(*n* = 36) and STAT (*n* = 32), group 4 contained sera samples of other febrile illness and PUO case (*n* = 35) and group 5 contained confirmed negative sera samples from healthy donors (*n* = 23).

**Result:**

The rOmp2a was found to be immunoreactive by iELISA and western blotting. The test showed a sensitivity of 93.75% and specificity of 95.83% when tested against 185 serum samples. For determination of statistical significance between experimental groups and control groups, Student’s *t* test was performed on the data.

**Conclusion:**

Omp2a emerges as a potential antigen candidate for serodiagnosis of human brucellosis.

## Background

Brucellosis is a major zoonotic disease and caused by various species of genus *Brucella*, a gram negative bacteria with 10 species namely *Brucella melitensis* (goat and sheep), *B. abortus* (cattle), *B. suis* (swine), *B. canis* (dogs), *B. ovis* (sheep), *B. neotomae* (desert mice), *B. cetacea* (cetacean), *B. pinnipedia* (seal), *B. microti* (voles) and *B. inopinata* (natural host unknown). The eleventh recently reported species of *Brucella* is from Baboon [1] but no infection has been reported till date. Among these the most pathogenic for human are *B. melitensis, B. abortus, B. suis,* and *B. canis* [2]. Brucellosis is a debilitating illness with more prevalence in western parts of Asia, India, Middle East, Southern European and Latin American countries and accounts for economic crisis as it causes veterinary morbidity and mortality [3]. Human brucellosis is characterized by weakness, fever, malaise, arthritis, osteomyelitis, endocarditis or meningoencephalitis [4] whereas in animals it causes chronic infection that leads to placentitis and abortion in pregnant females [5] and orchitis and epididymitis in males [6].

Although brucellosis is an endemic disease in many developing countries [7] but it is still under diagnosed or misdiagnosed. Brucellosis shows symptoms that mimic other febrile illness and eradication of the disease is possible only if it is accurately diagnosed and treated. The gold standard for diagnosis [8] is isolation from blood, tissue specimens, body fluids and bone marrow but serological tests forms the basis of diagnosis in most endemic regions. Among the various serological tests RBPT (Rose Bengal plate test) and complement fixation test are most widely used [9]. Cultural examinations suffer with drawbacks like they are time-consuming, hazardous and less sensitive [10]. Serological test are based on the detection of antibodies against LPS (lipopolysaccharide), but there is a high chance of cross reactivity as the LPS of *Brucella* shows resemblance to that of certain bacteria like *Y. enterocoloitica* O:9 and *E. coli.O:9* and *E.coli O157* [11]. New serological diagnostic tools that do not employ LPS are needed and several recombinant proteins were examined for serodiagnostic tests. Outer membrane proteins (Omp) are the non-LPS group of immunogens which can be a good replacement for vaccine and diagnostic purposes [12]. This study represents the cloning, expression and purification of Omp2a recombinant protein of *B. melitensis* in bacterial expression system and evaluation of its diagnostic potential with the clinical samples of human brucellosis.

## Methods

### Bacterial strains and cloning vector

*B. melitensis* 16 M strain, *B. abortus* S19 strain and *Y. enterocolitica* reference strains were obtained from National Collection of Type Cultures, United Kingdom and BR31, a human clinical isolate was isolated previously from blood culture. All bacterial strains were routinely maintained and cultured using Brain Heart Infusion (BHI) broth and agar medium (Himedia chemicals, Mumbai, India) under Bio Safety Laboratory Level 3 (BSL3) containment facility. The pET-SUMO expression vector used in the study for cloning of omp2a gene was procured from Invitrogen, CA, USA. The maintenance host *E. coli* strain Mach1 was obtained from Invitrogen and expression host *E. coli* strain BL21(DE3) was obtained from Sigma chemicals (St. Louis, MO, USA) (Table 1) and routinely cultured and maintained in Luria Bertani (LB) broth (Difco, Detroit, USA). The antibiotic selection was done by supplementing kanamycin (50 μg/mL) (Sigma) in LB broth and agar as a resistance marker for pET-SUMO vector.

**Table 1:** Details of vectors, gene primers and host cells used in this study

S.No.	Components used	Name/Sequence	Source
1	Vector	pET-SUMO	Invitrogen
2	Host cells	Mach1™-T1^R^ Chemically competent *E. coli* cells BL21(DE3) chemically competent *E*. *coli*. Cells	Invitrogen Sigma
3	Gene	omp2a	*B*. *melitensis* 16 M genome
4	Primer used for omp2a gene amplification	Forward - 5’ATGAACATCAAGAGCCTT3’ Reverse - 5’TTAGAACGAGCGCTG3’	This study
5	T7-promoter reverse sequencing primer of vector	Reverse - 5’TAGTTATTGCTCAGCGGTGG3’	Invitrogen

### Serum samples

A total of 185 clinical sera samples (suspected brucellosis cases of more than 15 days of recurring fever) were used which were obtained from different hospitals and laboratories of endemic regions of Karnataka, India collected during our previous study [13] and stored at DRDE, Gwalior. The sera samples were grouped into five groups, group 1 contained cultured confirmed positive for brucellosis (*n* = 15), group 2 contained sera samples of positive cases of brucellosis tested by conventional methods RBPT (*n* = 28) and STAT (*n* = 26) positive, group 3 contained sera samples which were RBPT (*n* = 36) and STAT (*n* = 32) negative, group 4 contained sera samples of other febrile illness and PUO case (*n* = 35) and group 5 contained confirmed negative sera samples from healthy donors (*n* = 23) (Table 2).

**Table 2:** Total numbers of serum samples grouped and evaluated

Group	Characteristics of samples	Total no. of samples
I	Culture confirmed positive cases of human brucellosis	15
II	RBPT positive + STAT positive (*n* = 28 + 26)	54
III	RBPT negative + STAT negative (*n* = 26 + 32)	58
IV	PUO cases of human samples	35
V	Confirmed negative cases of human brucellosis from healthy donors	23
	Total	185

### Cloning of omp2a gene in pET-SUMO vector

Omp2a gene (Accession No: CP007763.1 Region: 102,152–103,255) was PCR (polymerase chain reaction) amplified using the genomic DNA of *B. melitensis* 16 M strain. Genomic DNA was extracted from *B*. *melitensis* 16 M strain cultured overnight in BHI broth at 37 °C and 180 rpm (Shaker incubator, Labcon) using the DNeasy Blood and Tissue Kit (Qiagen, Germany) and checked on agarose gel electrophoresis for its purity and concentration was quantified using Nanodrop 2000 spectrophotometer (Thermo scientific). Primers (Forward Primer: 5’ATGAACATCAAGAGCCTT3’, Reverse Primer: 5′ TTAGAACGAGCGCTG 3′) used for amplification were designed using the software Gene runner and synthesized commercially. PCR conditions were optimized by performing temperature gradient PCR and standardized at initial denaturation of 95 °C for 5 min, 35 cycles of denaturation at 95 °C for 50 s, annealing at 54 °C for 50 s, extension at 72 °C for 1 min, followed by final extension at 72 °C for 10 min (Veriti, 96 well thermal cycler, Applied Biosystem). The bulk PCR product was then gel purified using Qiagen gel extraction kit (Qiagen,). The purified PCR product was also quantified using Nanodrop 2000 spectrophotometer and purity was checked by agarose gel electrophoresis. The purified product was then ligated into pET-SUMO vector and transformed into one shot Mach–T1 chemically competent *E.coli* cells. Positive clones were further screened by colony PCR with forward and reverse primers of the gene of clones obtained on LB agar plates supplemented with kanamycin (50μg/ml). The right orientation of the insert gene was confirmed by PCR performed by using forward primer of the gene and T7 reverse sequence primer of pET-SUMO vector. Plasmid isolation of the positive clones was done using QIAprep Spin Miniprep Kit (Qiagen) from overnight grown culture. Further expression of gene was done by transforming plasmid into BL21(DE3) chemically competent *E.coli* expression host cell. The final clones obtained on LB agar plates supplemented with kanamycin were then inoculated overnight into LB broth with kanamycin.

### Expression of the rOmp2a in BL21 (DE3) expression host cells

The above overnight grown culture was used for further inoculation in fresh LB broth. The expression of rOmp2a protein was done by induction with IPTG (isopropyl-β-D thiogalactopyranoside) using different concentrations (0.2 mM, 0.5 mM, 1 mM, 1.2 mM and 1.5 mM) and final concentration of induction was optimized with 1 mM IPTG (Sigma) at ~0.6 OD after 3 h of fresh inoculum of culture. The induced and un-induced fragments were collected at different intervals of 3 h, 5 h and overnight induction and further run on SDS-PAGE (12%) according to the protocol of Laemmli [14] (Fig. 2). To check whether the protein is soluble or forming inclusion bodies, 5 ml of induced pellet was dissolved in lysis buffer (50 mM NaH_2_PO_4_, 300 mM NaCl, 10 mM imidazole and 1 mg/ml of lysozyme). The lysed suspension were incubated on ice and then sonicated at 40 W amplitude for 10 min and 8 s of pulse (Vibra cell, Sonics, USA). The sonicated suspension was then centrifuged at 9600 x g for 10 min at 4 °C (Sorvall Legend Micro 21R Centrifuge, Thermo Scientific). The pellet and supernatant was collected and further checked for the expressed recombinant protein by SDS-PAGE analysis.

### Purification of rOmp2a protein under denaturing conditions

The expressed rOmp2a protein was found to be insoluble in pellet fraction and therefore purified under denaturing conditions using Ni-NTA affinity column chromatography. Purification was done employing a modified protocol reported earlier [15]. Briefly, 50 ml induced bacterial culture was pelleted and washed twice with 1X Phosphate Buffered Saline (PBS). The washed pellet was dissolved in 8 ml lysis buffer (8 M Urea, 100 mM NaH_2_PO_4_, 10 mM Tris–Cl, 20 mM β-mercaptoethanol, 1% Triton X-100, 1 mM phenylmethylsulfonyl fluoride (PMSF), pH 8.0) and sonicated for 10 min and kept at incubation of 1 h with continuous stirring at room temperature (27 °C) at 180 rpm. After 1 h incubation the lysed cell suspension was centrifuged at 12,000×g for 30 min at room temperature (RC5C, Sorvall, Thermo Scientific) and supernatant was collected and mixed with Ni-NTA agarose (Qiagen) in 1:3 ratio (Super-flow Ni-NTA slurry: Lysate) for 1 h to allow the rOmp2a 6X–His tagged protein to bind with Ni^2+^ in the super-flow slurry. The agarose super-flow Ni-NTA slurry-lysate mix was then packed in a 15 ml column and then flow-thru was collected. Further the column was washed with 20 ml wash buffer (8 M Urea, 100 mM NaH_2_PO_4_, 10 mM Tris–Cl, 1% Triton X-100, 10% glycerol, pH 6.3) to remove the unbound or weakly bound non-specific proteins. The rOmp2a porin protein was then eluted from the column using elution buffer (8 M Urea, 100 mM NaH_2_PO_4_, 10 mM Tris–Cl, pH 4.5) in 1 ml of 5 fractions and further analyzed on 12% SDS-PAGE. Refolding or dialysis of the purified porin protein was done against a decreasing urea gradient (8 M➝6 M➝4 M➝2 M➝1 M) and finally against 1X PBS. The concentration of purified rOmp2a porin protein was estimated by Bradford method using Bovine Serum Albumin (BSA) (Himedia) as standard [16].

### Experimental infection in mice with different Brucella strains

Bacterial strains of *B. melitensis* 16 M, a clinical isolate of *B. melitensis* 16 M (BR31), *B. abortus* S19 and *Y. enterocolitica* were cultured in 5 ml of BHI broth to experimentally infect the mice with live cells and to raise antisera against these strains. The 5 ml of each bacterial culture in BHI broth were serially diluted and plated on BHI agar to determine the CFU count. The 5 female Balb/c mice for each strains were grouped and infected with 2 × 10^5^ CFU/mice one group with 1X PBS as control. The blood was collected from infected and control mice after intervals of 15, 30 and 60 days post infection through orbital sinus of mice. The collected blood was incubated at room temperature for 1 h and then transferred to 4 °C for overnight. The sera was separated from the blood by centrifugation at 800 × g (Sorvall Legend Micro 21R Centrifuge, Thermo Scientific) for 10 min and stored at −20 °C until further use. All animal experiments were carried out in biosafety level 3 facility and had the approval of Institutional Animal Ethics Committee (No: 37/1999/CPC-SEA) and Institutional Biosafety committee wide protocol no: IBSC/15/MB/DTS/6 as per the institutional norms.

### Characterization of purified rOmp2a porin protein by Western blotting

Western blot analysis was done to characterize the rOmp2a porin protein using the protocol described by Towbin [17]. The protein was transferred from 12% polyacrylamide gel to nitrocellulose membrane (Pall Corporation, New York) using tris-glycine buffer supplemented with 20% methanol electrophoretically at 90 V for 45 min. After the transfer, membrane was blocked with 5% skimmed milk (Sigma) for 1 h at 37 °C. The membrane was then washed twice with 1X PBS containing 0.05% Tween (PBS-T) and final wash with 1X PBS alone with each wash of 5 min. The transferred membrane was cut into thin strips and 1 strip was incubated with an anti-His antibody conjugated to horseradish peroxidase (HRP) (Qiagen) in 1:1000 dilutions in PBS for 1 h at 37 °C. Washing steps were repeated as mentioned previously and membrane was developed with SIGMA FAST DAB (3′-3′-Diaminobenzidine tetrahydrochloride) tablets (cat. no. – D4293-50SET) in 20 ml distilled water.

### Sero-reactivity of rOmp2a

In order to check the sero-reactivity of rOmp2a porin protein, strips were incubated with antisera raised against *B. melitensis* 16 M strain in mice, a control mice with PBS only, a culture confirmed positive sera of brucellosis, a negative sera from healthy donor. The strips were incubated in 1: 50 dilution for 1 h at 37 °C and washing steps were repeated same as above. The strips were incubated with rabbit anti-human IgG conjugated with HRP (polyclonal antibody conjugate from Dako Cytomation, Glostrup, Denmark) at a 1:500 dilution for 1 h at 37 °C probed with human sera samples. This strips of *B. melitensis* 16 M sera raised in mice and control mice were incubated with anti mouse IgG HRP-conjugated polyclonal antibody (Dako Cytomation, Glostrup, Denmark). The strips were washed thrice same as above and then developed with SIGMA FAST DAB tablets in 20 ml water same as mentioned above. The membranes were finally washed 2 times with distilled water to stop the reaction.

### Evaluation of rOmp2a reactivity with sera raised in experimental infection in mice with different *Brucella* strains by indirect ELISA

Indirect microtitre plate IgG ELISA was standardized using the purified rOmp2a antigen. Protein was diluted to 25 μg/ml from the stock solution (126 mg/L) in 1 M carbonate bicarbonate buffer (pH 9.6) and 100 μl of this solution was coated on to Nunc immunoplates. Plates were then incubated at 37 °C for 1 h and washed thrice with PBS-T for 5 min each. The plates were then blocked with 100 μl of 1% BSA in 1X–PBS and incubated at 4 °C for overnight. After overnight incubation plates were again washed with PBS-T thrice and stored at 4 °C until used. The mice sera collected at different time intervals were diluted in 1:100 in PBS and 100 μl was added to per wells into plates and incubated at 37 °C for 1 h and washing steps were repeated thrice. Plates were then incubated with anti-mice IgG HRP-conjugated (1:1000 dilutions) for another 1 h at 37 °C and then washed. The plates were developed with SIGMA FAST OPD (o-phenylenediamine dihydrochloride) tablets (cat. no. - P9187-50SET) and kept in dark for 5 min for colour development. The reaction was stopped by the addition of 30 μl of 0.5 M H_2_SO_4_ per well and the absorbance was read at 490 nm in a spectrophotometer ELISA reader (μQuant, BioTek, US). To ensure uniformity in testing all samples were run in duplicate and the mean absorbance value was used for calculating final OD of the each samples.

### Evaluation of rOmp2a porin antigen for diagnosis of brucellosis with clinical samples by indirect ELISA

Indirect microtitre plate IgG ELISA was performed with 185 clinical serum samples collected from regions endemic to brucellosis from southern part of India. The samples were grouped into five groups. Group 1; cultured confirmed positive serum samples of brucellosis (*n* = 15), group 2; sera samples from positive cases of brucellosis previously tested by conventional methods of RBPT (*n* = 28) and STAT (*n* = 26), group 3; sera samples negative by RBPT(*n* = 36) and STAT (*n* = 32), group 4; sera samples of other febrile illness and PUO case (*n* = 35) and group 5; confirmed negative sera samples from healthy donors (*n* = 23).The protocol followed for iELISA was the same as discussed above but instead of mice sera and HRP-conjugated anti-mice IgG, human clinical sera samples and rabbit anti-human IgG conjugated with HRP secondary antibody were used.

### Statistical analysis

For determination of statistical significance between experimental groups and control groups, Student’s *t-*test was performed on the data. A *P* value of ≤0.05 was considered significant.

## Results

### Characterization of recombinant construct of Omp2a in pET-SUMO

**Fig. 1:**
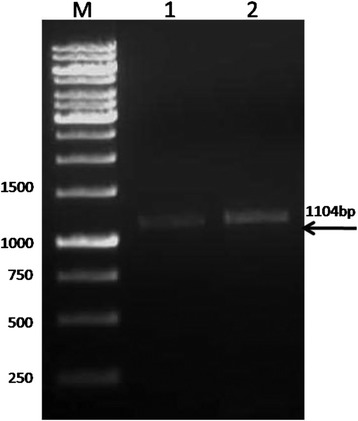
PCR amplification of Omp2a gene. *Lane M* - 1 kb DNA ladder (#SM0313, Fermentas), *Lane 1* – Omp2a PCR amplified, *Lane 2* - Omp2a PCR amplified

The rOmp2a porin gene was PCR amplified and 1104-bp (Fig. 1) gel purified product was ligated into a pET-SUMO expression vector. The ligated product transformed initially into Mach1™^-T1R^ Chemically competent *E. coli* cells and colonies were grown on the LB agar media plates supplemented with kanamycin were screened and selected for the presence of insert by rOmp2a gene PCR. A total of 7 colonies were found positive by PCR when they were checked for correct orientation of insert by using forward primer of the gene and reverse primer T7-promoter of pET-SUMO vector. Four clones showed the correct orientation out of which 1 clone were selected for protein expression analysis.

### Expression analysis and purification of rOmp2a porin protein

**Fig. 2:**
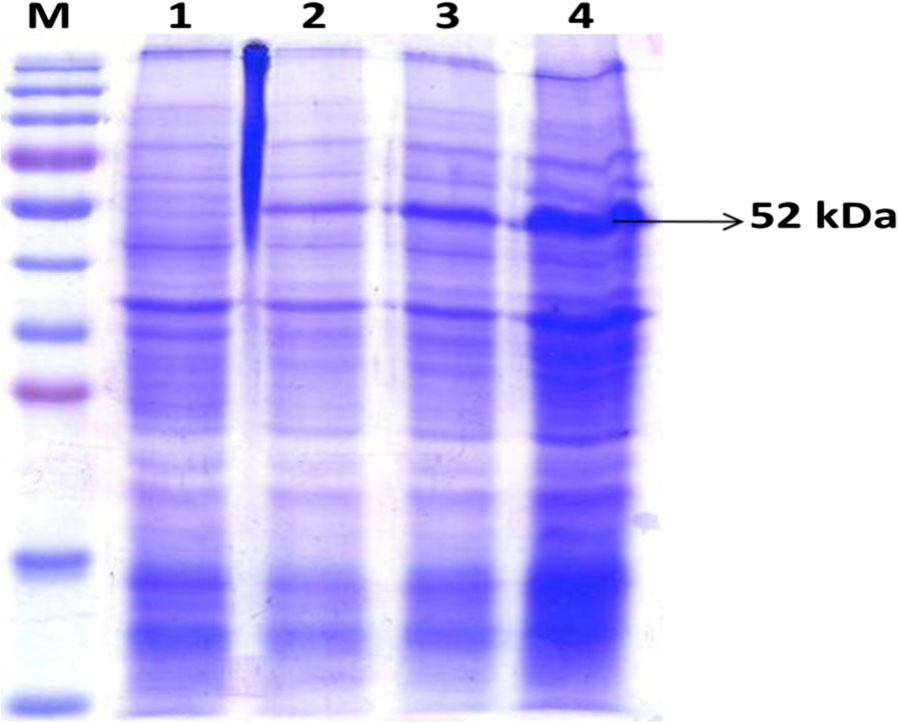
Expression of rOmp2a protein. *Lane M* – Pre-stained Protein marker (#PG-PMT2922, PUREGENE) *Lane 1* – uninduced 3 h, *Lane 2* – induced 3 h, *Lane 3* – induced 5 h, *Lane 4* – induced overnight

**Fig. 3:**
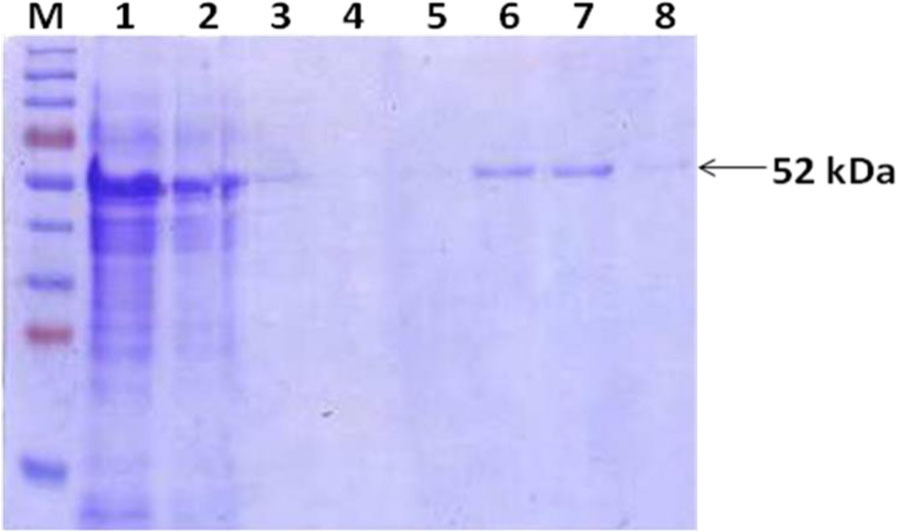
Purification of rOmp2a under denaturing conditions. *Lane M* – Pre-stained marker (#PG-PMT2922). *Lane 1*- Clear lysate, *Lane 2*- Flow thru, *Lane 3*- Wash 1, *Lane 4* – Wash 2, *Lane 5* – Wash 3, *Lane 6* – Elute 1, *Lane 7* – Elute 2, *Lane 8* – Elute 3

The selected clone was expressed rOmp2a protein along with fusion protein of vector at 52 kDa (41 kDa targeted omp2a + 11 kDa fusion protein of pET-SUMO vector) when visualized on 12% SDS-PAGE. The expression conditions were finalized with 1 mM IPTG concentrations induced at 0.6 OD and post induction for overnight expression of protein of maximum yield (Fig. 2). The protein was found to form inclusion bodies and purification conditions were optimized with 8 M urea under denaturing conditions. Wash buffer (pH 6.3) with additionally containing 10% glycerol and 1% triton-X 100 helped in the removal of non specific proteins whereas the recombinant Omp2a porin protein was eluted from the column by further lowering the pH of elution buffer (pH 4.5). The different fractions of washes and elutes were analyzed by SDS-PAGE showing the purity of 95% of rOmp2a protein (Fig. 3). Purified rOmp2a porin protein was then refolded by dialyzing against urea gradient of 6 M, 4 M, 2 M, 1 M and PBS. The final concentration of the dialyzed and pooled recombinant protein was found to be 126 mg/L estimated by Bradford protein estimation assay.

### Characterization of purified rOmp2a protein by Western Blotting

**Fig. 4:**
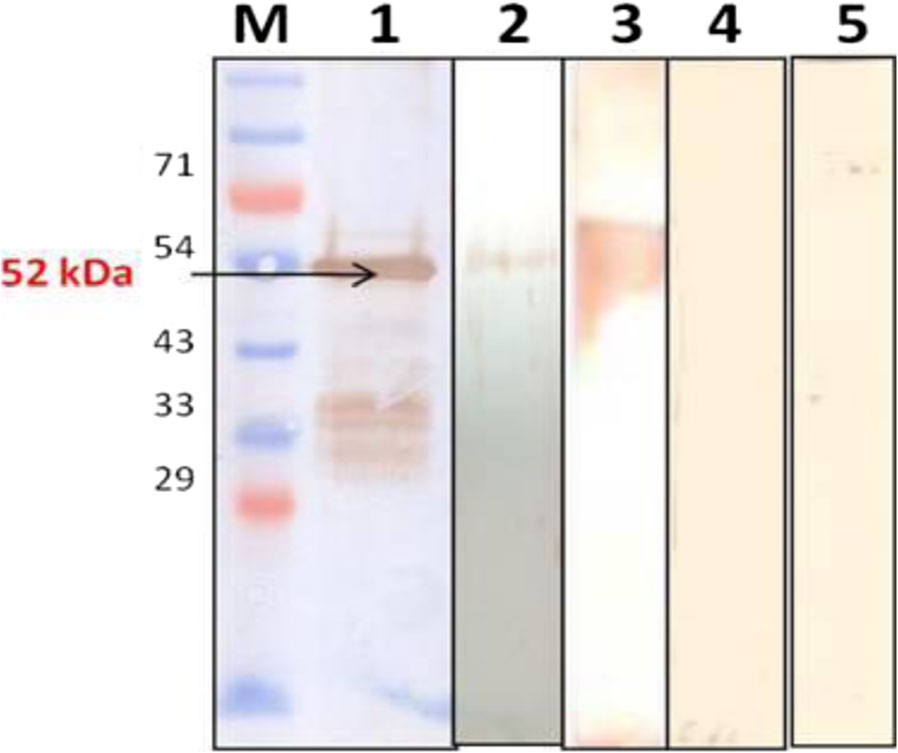
Reactivity of rOmp2a antigen by western blot. *Lane M* – Pre-stained Protein marker (#PG-PMT2922, PUREGENE), *Lane 1* – anti His Antibody, *Lane 2* – positive sera of culture confirmed clinical sample of human brucellosis, *Lane 3* – antisera of *B. melitensis* 16 M reference strain raised in mice, *Lane 4* – confirmed negative sera from healthy donor, *Lane 5* – antisera of control mice with PBS only

The western blotting of purified rOmp2a porin protein was done with anti-His-HRP antibody, two samples of culture confirmed positive sera of human brucellosis, antisera raised against *B*. *melitensis* 16 M reference strain in mice, human sera from confirmed negative sample and antisera from control mice. A colored band was seen as reactivity only with the anti-His-HRP antibody, culture confirmed positive samples of human brucellosis and antisera raised against reference strain of *B*. *melitensis*. Whereas no reactivity was seen with the control mice sera, negative sample of brucellosis and sample from healthy donor (Fig. 4).

### Evaluation of rOmp2a reactivity with sera raised in experimental infection in mice with different *Brucella* strains by indirect ELISA

**Fig. 5:**
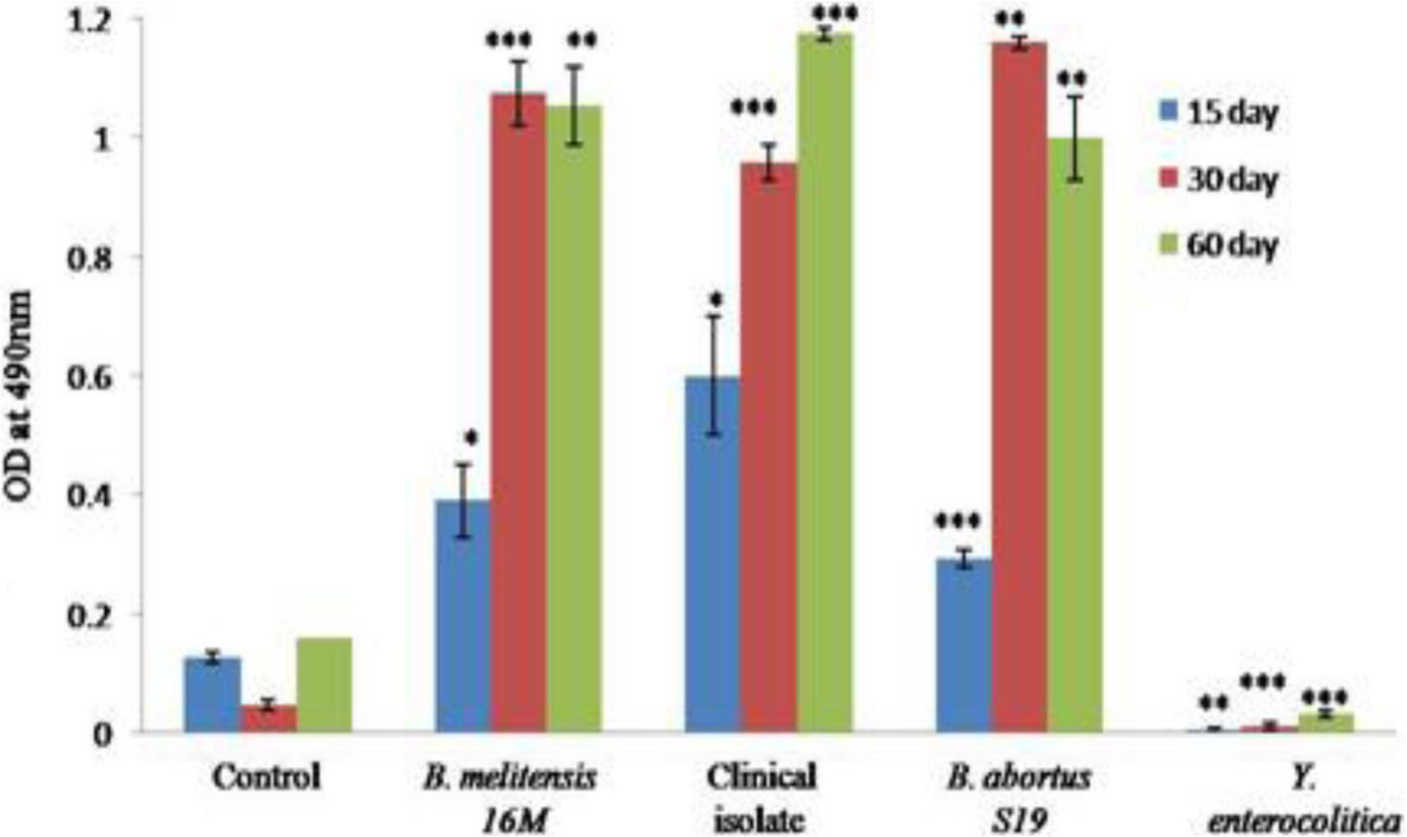
Reactivity of rOmp2a with sera from experimental infection in mice. Reactivity of rOmp2a with sera from mice experimentally infected with *B. melitensis 16 M*, *BR31* (Human clinical isolate), *B. abortus S19* and *Y. enterocolitica,* and control mice treated with PBS only. Blood was collected from all groups after 15, 30 and 60 days. Data are presented as the *A*490 of a 1:100 dilution of serum. Each *bar* indicates the average standard deviation of triplicate samples. *Asterisk* indicates statistical significance, determined by Student’s *t* test. A *P* value of _0.05 was considered significant (*P < 0.05; **P < 0.01. ***P < 0.001)

To check the reactivity of rOmp2a porin protein it was tested against sera collected from experimentally infected mice with *B. melitensis* 16 M strain, a clinical isolate *B. melitensis* (BR31), *B. abortus* S19 strain and *Y. enterocolitica* strain*.* The rOmp2a protein was found to be reactive in iELISA with sera against *B. melitensis* 16 M, clinical isolate BR31 and *B. abortus* S19 but showed no reactivity with sera collected with control mice. To check the cross reactivity of this recombinant protein it was also checked against *Y. enterocolitica* experimentally infected sera and showed no reactivity (Fig. 5).

### Evaluation of diagnostic potential of rOmp2a with clinical samples by iELISA

**Fig. 6:**
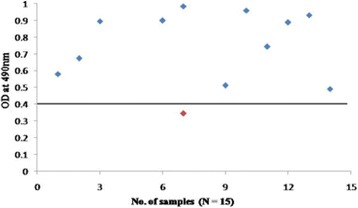
rOmp2a antigen iELISA with culture confirm positive sera samples

**Fig. 7:**
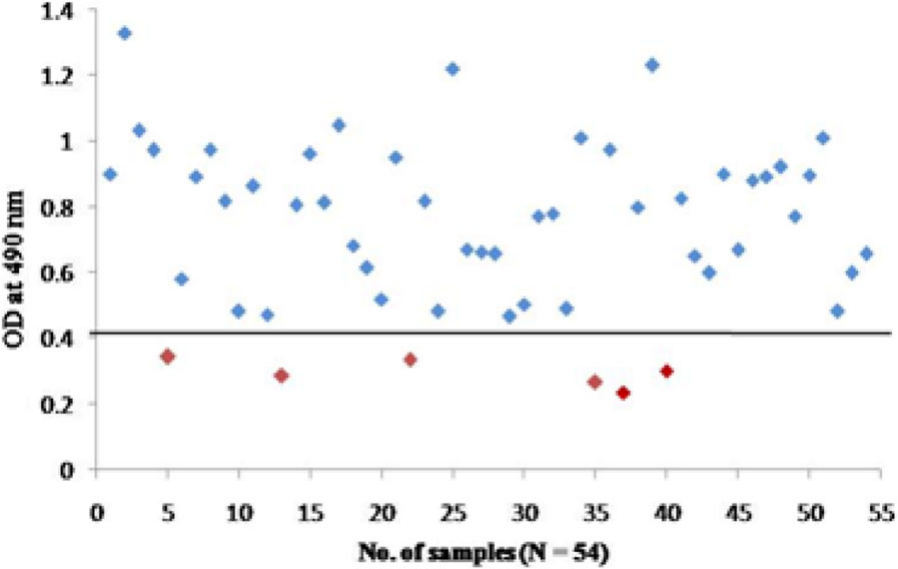
rOmp2a antigen iELISA with RBPT and STAT positive sera samples

**Fig. 8:**
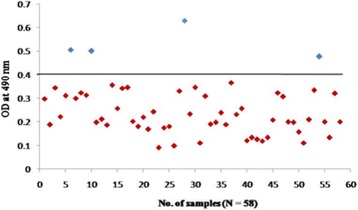
rOmp 2a antigen iELISA with RBPT and STAT negative sera samples

**Fig. 9:**
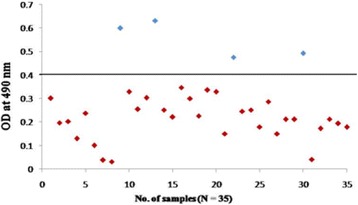
rOmp2a antigen iELISA with other febrile illness and PUO cases sera samples

**Fig. 10:**
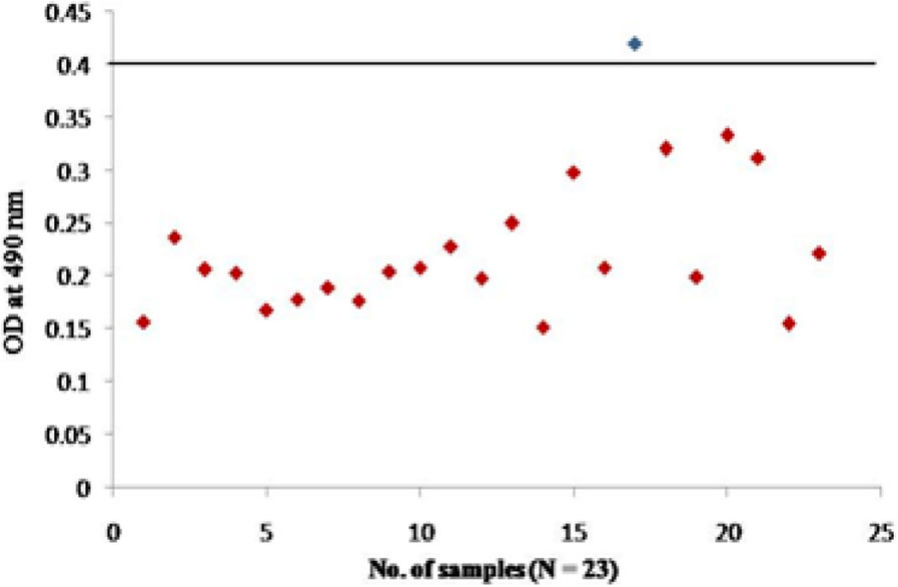
rOmp2a antigen iELISA with confirmed negative sera samples from healthy donors

To confirm further the reactivity of rOmp2a porin protein and its potential use in diagnosis of human brucellosis 185 sera samples were tested by indirect plate ELISA. group 1 included culture confirmed positive sera of brucellosis (*n* = 15) out of 15 samples 14 was found to be positive by rOmp2a porin protein as antigen and 1 as negative (Fig. 6), in group 2 out of 54 sera samples positive by both RBPT (*n* = 28) and STAT (*n* = 26), in RBPT (*n* = 28), 23 samples were picked up as positive by rOmp2a iELISA and 5 samples were found to be negative by iELISA. In STAT (*n* = 26), 25 samples were found to be positive and 1 sample as negative by iELISA (Fig. 7). Group 3 included samples negative by RBPT (*n* = 26) and STAT (*n* = 32), 1 sample from RBPT and 3 from STAT were found to be positive by rOmp2a iELISA whereas 25 and 29 samples were found to be negative by iELISA from RBPT and STAT respectively (Fig. 8), group 4 consist of 35 samples of other febrile illness out of which 2 samples were found to be positive by rOmp2a iELISA and 33 samples found to be negative by rOmp2a iELISA (Fig. 9). Group 5 contained 23 confirmed negative sera samples from healthy donors, out of 23 samples 1 was picked up as positive and 22 samples were found to be negative by rOmp2a porin antigen iELISA (Fig. 10). The cut off value for iELISA was calculated as OD value greater than 0.407 (Table 3). It was calculated as the mean OD value (0.227) plus three Standard Deviations (±0.06) of confirmed negative samples (healthy donors). Samples which were positive by culture were defined as true positive and the sera samples obtained from the healthy donors were considered as true negative. Samples which were culture positive but were found to be negative by the iELISA were considered as false negative and samples from healthy donors found to be positive by conventional or the rOmp2a porin antigen iELISA were considered as false positive. The sensitivity and specificity of the rOmp2a porin iELISA was calculated and found to be 93.75% and 95.83% respectively. Correlations of rOmp2a porin iELISA with RBPT and STAT was also calculated and found to be 94.44% and 96.55% respectively. The likelihood ratio for positive and negative results was estimated to 22.3 and 15.2 respectively (Table 4).

**Table 3:** Performance of iELISA using rOmp2a antigen

S.No.	Group	Positive by iELISA	Negative by iELISA
1	I	14(93.3%)	1(6.66%)
2	II	23(82.1%), 25(96.1%)	5(17.8%), 1(3.8%)
3	III	1(3.8%), 3(9.37%)	25(96.1%), 29(90.6%)
4	IV	2(5.7%)	33(94.2%)
5	V	1(4.3%)	22(95.6%)

**Table 4:** Sensitivity and Specificity of iELISA using rOmp2a antigen

S.No.	Calculations	Porin 2 antigen
1	Senstivity(%)^a^	93.75
2	Specificity(%)^b^	95.83
3	Correlation with RBPT(%)^c^	94.44
4	Correlation with STAT(%)^c^	96.55
5	Likelihood ratio for positive result^d^	22.3
6	Likelihood ratio for negative result^e^	15.2

^a^Sensitivity = [true positives/(true positives + false negatives)] × 100

^b^Specificity = [true negatives/(true negatives + false positives)] × 100

^c^Correlation = [number of samples positive by both methods + number of samples negative by both methods]/[total number of samples] × 100

^d^Likelihood ratio for positive result = sensitivity/1 − specificity

^e^Likelihood ratio for negative result = specificity/1 − sensitivity

## Discussion

Brucellosis is an emerging zoonotic infection and is endemic in many parts of the world including India. Early and accurate detection of *Brucella* spp. is important for adequate treatment of brucellosis since misdiagnosis can lead to high case fatality rate [18]. The current gold standard for the laboratory based diagnosis of brucellosis is based on isolation of bacteria from clinical samples which require long cultivation periods, risk of laboratory acquired infection and culture isolation rate is very low in most cases [19]. Because of these limitations the need of serological and molecular diagnosis is considered as the best alternative for brucellosis diagnosis. Serodiagnosis is given importance in most endemic areas as the test can be performed easily in limited clinical set up with minimum resources in comparison to molecular techniques. The antibody response to *Brucella* consists of the early production of IgM and immediate progress to the production of IgG2 and small amount of IgG1 [20]. *Brucella* a facultative intracellular pathogen resides and multiplies in macrophages but once it has completed its intracellular cycle it is released from the cells by lytic and non lytic mechanism and new release *Brucellae* induces the humoral response of IgG antibodies. Presently the serodiagnosis of brucellosis is based on detection of anti LPS antibodies but it requires growth of bacterial culture in laboratory and associated risk to laboratory persons which are handing the *B*rucella species*.* The LPS of *Brucella* species shows high similarity with other bacterial species like *Y. enterocolitica, E. coli, Salmonella species* etc. and thus have a chance of cross reactivity with related bacterial species and hence less specific*.* Therefore there is an urgent need to develop the diagnostic system based on recombinant antigens and several reports are available based on Outer membrane proteins (Omps) as the antigen of choice to be used in sero diagnosis [12].

Omps being the major immunoreactive components in the bacterial cells and hence has received the most attention as antigen candidates for development of new diagnostic assays. *Brucella* Omps, have been classified according to their molecular masses; group 2 porins (36-38kda) and group 3 proteins (25-27kda) [21]. Omps plays an important role in the virulence factors of *Brucella* and the diagnostic potential of various Omps of *Brucella* species have been studied by various groups. Brucella Omp31 has been identified as an interested candidate for serodiagnosis of *Brucella* infections in rams [22] and in farm goats and sheep having history of abortions induced by *B. melitensis* [23]. Omp28 protein was also found to be highly reactive with brucellosis positive sera of human clinical samples [24]. In another study, Omp28 also showed reactivity to *Brucella* infected cattle, dog, sheep, and goat sera [25]. Comparative evaluation of the recombinant omp28 and omp31 for diagnosis of human brucellosis by ELISA has also been studied and rOmp28 antigen was found to be more suitable antigen for the clinical diagnosis of brucellosis [26]. The combined use of the recombinant *B. abortus* Omp10, Omp19 and Omp28 proteins for the clinical diagnosis of bovine brucellosis is also reported [27]. The role and size of each Omps is various but the porin Omps being the bacterial components have important roles in the bacterial survival [28]. In *Brucella* species, Group 2 Omps proteins are identified as porins and among them Omp2a and Omp2b are 39 kDa general diffusion pores with 85% sequence identity. In a study, the immunogenicity of *B. abortus* Omp2b has been investigated to discover LPS free protein as new diagnostic candidate. Recombinant Omp2b (rOmp2b) elicited the production of TNF-α and IL-6 from the RAW 264.7 cells after 24 h stimulation. In in-vitro stimulation of rOmp2b, spleen cells from naive mice produced high levels of IFN-γ and low levels of IL-4. In in-vivo rOmp2b immunized mice also produced antigen-specific IgM and IgG antibodies in high titre [29]. Various studies to investigate gene expression of porins proteins under environmental conditions directly associated with *Brucella* pathogenicity have also been completed. Thus rOmps and porin proteins are associated directly with the pathogenicity of *Brucella* species and high immunogenicity of rOmp2b is reported. The present study was conducted to evaluate another associated porins protein of *Brucella melitensis* 16 M. In order to study Omp2a as candidate antigen for serodiagnosis of *Brucella* species, the gene of Omp2a was cloned in pET-SUMO prokaryotic expression vector, and the protein was expressed using BL21 DE3 host cells. The expressed recombinant protein was purified and tested in iELISA format for its reactivity with antisera collected from experimentally infected mice at different time intervals of 15, 30 and 60 days. The antigen showed high reactivity with *B. melitensis* 16 M, clinical isolate of *B. melitensis* 16 M and *B. abortus* S19 sera whereas it showed no reactivity with *Y. enterocolitica*. Although *B. abortus* S19 vaccine strain for animal but can cause infection in human and reactivity of rOmp2a with antisera of *B. abortus* S19 strain showed importance of rOmp2a porin protein in animals acquired infections of human brucellosis. Later, it was tested against well defined clinical sera samples divided into 5 groups. The sensitivity and specificity of the rOmp2a porin iELISA was found to be 93.75 and 95.83% respectively. The correlations of rOmp2a porin iELISA with RBPT, STAT, and RBPT + STAT was also calculated and found to be 94.44 and 96.55% respectively. The high sensitivity and specificity of rOmp2a indicate its potential as an important candidate antigen for serodiagnosis of brucellosis.

## Conclusion

The rOmp2a is a potential antigen for serodiagnosis of human brucellosis. The efficacy of serodiagnosis of also needs to be further evaluated on several sets of well defined sera samples from different geographic regions on large number of clinical samples. This will further establish the use of rOmp2a as for serodiagnosis for human brucellosis. The comparative evaluation for rOmp2a iELISA with other reported recombinant Omps (Omp 28, Omp 31 etc.) and also their combinational use can also be explored. In addition, the evaluation of rOmp2a antigen for serodiagnosis of bovine brucellosis can also be evaluated.

## Abbreviations

BHI: Brain Heart Infusion
BSA: Bovine serum albumin
DAB: 3′-3′- Diaminobenzidine tetrahydrochloride
HRP: Horseradish peroxidase
IPTG: Isopropyl-β-D thiogalactopyranoside
LB: Luria Bertani
LPS: Lipopolysaccharide
Omps: Outer membrane proteins
OPD: Ortho-phenylenediamine dehydrochloride
PBS: Phosphate buffer saline
PBST: Phospahte buffer saline tween
PCR: Polymerase chain reaction
PMSF: phenylmethylsulfonyl fluoride
RBPT: Rose Bengal plate test
STAT: Standard tube agglutination test
